# The Hospital

**Published:** 1918-01-26

**Authors:** 


THE
### INSTITUTIONAL WORKER
**Being a Special Supplement to "The Hospital"**

### OUR BUREAU OF INFORMATION.

### Rules for Correspondents.

1. Every letter must be accompanied by the coupon to be cut from the back cover (inside page) of THE HOSPITAL, current issue, and must contain the name and address of the correspondent with pseudonym for publication if desired. Replies by post cannot be given save under exceptional circumstances at the Editor's discretion.

2. Letters from Approved Homes in reply to special needs published in the Bureau should state terms and full particulars, and be sent prepaid under cover to the Editor of the Bureau with name written across coupon for identification.

3. Proprietors of Homes which have not yet been entered on the List of Approved Homes, but have spare accommodation likely to suit special needs, are invited to write for an application form for registration. The fee for registration, which includes two announcements of the Home in the Bureau and other privileges, is 10s.

4. All communications to be addressed to the Editor of THE HOSPITAL, 28 Southampton Street, Strand, London, W.C. 2, and marked "Bureau of Information."

### INSTITUTION FACTS AND FIGURES.

### Stiff Joints and Other Injuries.

#### Simple Apparatus for their Treatment.

AMONGST the many new and improved inventions brought forward during the war for the treatment of stiff joints, the simple and easily-made apparatus invented and used by Mrs. Guthrie-Smith, A.P.M.M.C., at the Seaford Command Depôt, are well worth studying, especially in the case of soldiers who, though not able to return to the Front, are fit for light duties. A feature in connection with this apparatus which especially merits attention is that its use brings into play mental as well as physical power, but as the former is not immediately directed to the movement accomplished, physical resistance is lessened. It is also claimed that a greater variety of movements can be obtained than is possible by manual manipulation.

The inventor has adopted the term "strafe" (by which name the soldiers under treatment always refer to it) as the general name of the apparatus. The foundation for the pieces of apparatus required for exercising various parts of the body consists of a framework or gallows fitted with cord and cleats for strap attachments, with a movable post in slots to allow for adjustment to all sizes of patients and the various "strafes."

THE SHOULDER STRAFE.

This consists of a harness belt over the clavicle and another over the scapula, the clavicle belt being fixed to the floor of the framework by means of a cord and cleat, and the belt going round attached to the side of the frame. If the right shoulder is being treated, the left foot should be placed on a block and the knee of that leg protected by a pro-plastic splint to prevent any injury accruing from that position, and for comfort. The patient than grasps the stirrup handles at the end of the two pulleys attached to the top of the frame and through the movable post. In this position the patient throws the whole of his weight on to the axillary border of the scapula. The injured arm should be rotated out and to the back of the post. Both passive and active movements can be obtained in accordance with the adjustment of weights. The patient, when placed in the proper position, should then work the pulleys in an upward and downward movement.

The principal points to note in the use of this apparatus are (1) the adjustment of ropes and harness, (2) good sloping position of the patient on to his own scapula, (3) the possibility of fixing all bones except the humerus, (4) the voluntary effort of the patient to his own cure, (5) the amount of "rotation out" which is possible and so necessary to an angle of 90 degrees —viz. ¬—is sufficient with the clavicle fixed.

HIP STRAFE.

One of the advantages that is claimed for this "strafe" is that the "hamstring" contractions can be outstretched with a few ounces of strength as compared with the usual muscular effort required. The patient is fixed to a couch inclined downwards by a broad belt (the couch should be raised 8 inches at the head), and the pulley which is attached to the frame or gallows should just clear the patient's shoulders. The wounded leg should be protected by a poro-plastic splint to prevent the knee outstretching, and if a knee-cap with straps is adjusted over the knee, a good degree of straightening in the joint can be obtained. The patient should wear stiff canvas shoes with loop at toe.

MOVEMENT.—The patient alternately raises and lowers both legs over the pulley and short rope, counting slowly. The operator can, if desired, overstretch the leg at the end of each completed movement. The double action of the patient tends to overcome his natural resistance; he is also making a voluntary effort to cure himself.

The principal points to observe in the use of the hip strafe are (1) the mechanical disadvantage of the patient; (2) the force of gravity employed; (3) short rope joining legs in first position; (4) exercise consists of lowering one leg against the other; (5) protection of wounded leg against over-straightening; (6) knee-cap to straighten joint; (7) possibility of easy over-straightening of patient at close of each leg movement.

Other "strafes" are the elbow strafe and the knee strafe, for which the same pulley as used in the hip strafe can be utilised, the couch ending at the knee.

Mrs. Guthrie-Smith gave an interesting demonstration of her home-made appliances under the auspices of the Incorporated Society of Trained Masseuses, at 3 Vere Street, W., recently.

The apparatus can be obtained from the Medical Supply Association, Gray's Inn Road, W.C., for £10, and an angle-measurer, such as used by Mrs. Guthrie-Smith, for 14s.

**The Editor will be glad to receive correspondence and to consider contributions upon all subjects relating to institutional work which affect the welfare of institutional workers.**

### JANUARY QUESTIONS.

**1. Extending a Hospital Engineer's Work.**

In what ways can a hospital engineer be useful in addition to his routine work of attending to the heating apparatus?

**2. A Domestic Matter in a Nurses' Home.**

Describe the cleanest, quickest, and most careful method of washing and drying crockery in a nurses' home to accommodate 180 persons.

### RULES.

The following rules must be observed:—
1. Contributions must be written on one side of the paper. Brevity and terseness are desirable features. The MSS. must bear the name and address of the sender and be accompanied by coupon to be cut from the back cover (inside page) of the current issue of The Hospital. A pseudonym must be chosen if the name is not to be published.
2. Contributions must be addressed to the Editor of The Hospital, Institutional Worker Supplement, 28 & 29 Southampton Street, Strand, London, W.C. 2, should reach him before the end of the current month, and be marked in the left-hand corner "Facts and Figures."

**A minimum payment of Five Shillings will be made for each published answer to the January Questions.**

### QUESTIONS INVITED.

In connection with our Question Box, we offer every month five shillings each for the two best questions which are sent in for consideration. The questions may be on any institutional subject and concern any department, from the secretary's to the porter's, or the matron's to the domestic staff; the one essential is that they must be practical and deal with points that have a definite relation to hospital or institutional administration, upkeep, finance, or management. Points relating to artificers' work, housekeeping, the laundry, out-patients, and other practical matters will be welcome. It is hoped that thus, when difficult or doubtful points arise in the course of their work, institutional workers will be encouraged to put them in the form of a question and send them to the Editor, The Hospital Bureau of Information, 28 Southampton Street, Strand, W.C. 2, marked "Question Box."

The best questions will be published in due course and our readers will be given the opportunity of answering them. Thus all institutional workers may be able to co-operate with the view of helping the work and smoothing the difficulties of each other. In short, we want the Question Box of the *Institutional Worker* to be freely used by every worker habitually.

### ENQUIRIES AND ANSWERS.

### *THE SICK AND IN NEED.*

**Treatment for Rheumatism.**

Write, giving full particulars, to the secretaries of the Devonshire Hospital, Buxton, and of the Alexandra Hospital, Woodhall Spa, who will doubtless tell you if you are eligible for admission to their institutions. Enclose stamped addressed envelopes for replies.—Sick One.

### *WAR MATTERS.*

**Knitted Comforts for Troops in the Field.**

The Director-General of Voluntary Organisations is appealing for mufflers, helmets, and other knitted articles for the troops in the field, as well as pyjamas, slippers, dressing-gowns, and hospital bags for military hospitals. As you state that you have several of the articles named which you wish to give away, we suggest that you send them addressed to your nearest Voluntary Organisations Depôt or to the Comforts Depôt, 45 Horseferry Road, Westminster, S.W. 1.—Worker.

**Fee for Certificate for Non-Members of V.A.D.s.**

In future a fee of 2s. 6d. will be charged to non-members of the Society's detachments for each certificate issued to them after passing the Society's examinations. The fee will be payable to the Secretary, British Red Cross Society, 83 Pall Mall, S.W. 1.—Non-Member.

**Departments of Joint War Committee.**

The different departments of the Joint War Committee and the British Red Cross Society situated at 83 Pall Mall, London, S.W. 1, are: Accountants; Auxiliary Red Cross Home Hospitals; British Red Cross Hospital, Netley, Committee; Central Joint V.A.D. Committee; Church Collections; Collecting Boxes; Collections Committee; Contracts; Convalescent Camps; Convalescent Homes (Officers); Hospital Trains; Inoculation; Joint War Committee Compassionate Fund; Joint War Committee; Joint War Finance Committee; Motor Ambulances; Motor Launch and Hospital Ship; Orderlies; Organising Secretary, and Secretary of British Red Cross Society; Personnel (Doctors); Personnel and Nursing Organisation; Star and Garter Committee; Stores and Transport; Trained Nurses; Travelling and Transports.—E. G. B.

### *EMPLOYMENT AND TRAINING.*

**Training at Eye Hospitals in Liverpool.**

You might apply to the matron of each of the two following institutions for a vacancy: Liverpool Eye and Ear Infirmary, Myrtle Street, Liverpool; applicants are received for two years' training after a two months' trial period, at the age of twenty-one, and receive a salary of £10 the first year and £14 the second. At the St. Paul's Eye Hospital, Oldhall Street, Liverpool, candidates are received at the age of nineteen for a two years' course of training at a salary of £12 and £14 the first and second years respectively.—S. O.

**Training as Hospital Nurse.**

We are afraid you are too young to begin training in a large general hospital, even the small institutions seldom receive probationers under twenty-one years of age. Would you care to take a course of training in the nursing of sick children? Or you might get some preliminary training at a cottage hospital until you are old enough for general training. Watch the advertisements which appear in the nursing journals from week to week; also obtain "How to Become a Nurse," published by The Scientific Press, Ltd., 28 and 29 Southampton Street, Strand, W.C. 2, price 2s. 10d. post free, which contains a full list of institutions and much other useful information.—T. H. P.

### *MISCELLANEOUS.*

**Cost of X-Ray Installation for a Small Hospital.**

An instructive and interesting article upon the "Cost of *X*-ray Installation for a Small Hospital" appeared in the columns of the *Institutional Worker* in The Hospital for November 8, 1913. Of course, it must be borne in mind that all *x*-ray apparatus has increased 25 per cent. in cost since the outbreak of war.—Hospital Manager.

**An Ideal Crutch.**

Among the numerous types of crutches which have come before our notice—and there have been many notable improvements in recent times, the one which in our opinion takes a high place is the Harley Hammock Crutch. It is an ideal crutch, and by its use any possible danger of crutch paralysis, abscesses, or sore hands may be obviated. On each crutch a light framework is fitted, from which a specially constructed seat or saddle is suspended, adjustable to suit the height of the patient. Flexibility is ensured by adjustable springs, and jarring of sensitive nerves is thus prevented. The light framework at the top of the crutch is fixed on a pivot from which the hammock saddle is hung so that the whole of this suspended portion swings with the body. It will thus be clear that the combination provides a seat of which the patient can avail himself while walking, and that pressure under the axilla and strain upon the hands is avoided. The apparatus can be fitted to existing crutches of suitable design. The manufacturers are A. L. Gifkins and Co., Ltd., 68 Victoria Street, S.W. 1.—Commandant.

